# Proper 5’-3’ cotranslational mRNA decay in yeast requires import of Xrn1 to the nucleus

**DOI:** 10.1371/journal.pone.0308195

**Published:** 2025-01-22

**Authors:** Antonio Jordán-Pla, Yujie Zhang, José García-Martínez, Shiladitya Chattopadhyay, Anabel Forte, Mordechai Choder, Vicent Pelechano, José E. Pérez-Ortín

**Affiliations:** 1 Facultad de Biológicas, Instituto de Biotecnología y Biomedicina (BIOTECMED), Universitat de València, Burjassot, Spain; 2 Department of Microbiology, SciLifeLab, Tumor and Cell Biology, Karolinska Institutet, Solna, Sweden; 3 Rappaport Faculty of Medicine, Department of Molecular Microbiology, Technion-Israel Institute of Technology, Haifa, Israel; 4 Facultad de Matemáticas, Departamento de Estadística e Investigación Operativa, Universitat de València, Burjassot, Spain; Western University, CANADA

## Abstract

The budding yeast Xrn1 protein shuttles between the nucleus, where it stimulates transcription, and the cytoplasm, where it executes the major cytoplasmic mRNA decay. In the cytoplasm, apart from catalyzing 5’→3’ decay onto non translated mRNAs, Xrn1 can follow the last translating ribosome to degrade the decapped mRNA template, a process known as “cotranslational mRNA decay”. We have previously observed that the import of Xrn1 to the nucleus is required for efficient cytoplasmic mRNA decay. Here by using an Xrn1 mutant that cannot enter the nucleus, but is otherwise functional in ribonuclease activity, we show that nuclear import is necessary for proper global cotranslational decay of mRNAs along coding regions and also affects degradation in the of 5’ region of a large group of mRNAs, which comprise about 20% of the transcriptome. Furthermore, a principal component analysis of the genomic datasets of this mutant and other Xrn1 mutants also shows that lack of a cytoplasmic 5’→3’ exoribonuclease is the primary cause of the physiological defects seen in a *xrn1Δ* mutant, but also suggests that Xrn1 import into the nucleus is necessary for its full *in vivo* functions.

## Introduction

*Saccharomyces cerevisiae* Xrn1 (also known as Kem1) [1] is a 5’→3’ exoribonuclease that is highly conserved among eukaryotes and is responsible for mRNA 5′→3′ exonucleolytic decay [2–4]. It also plays additional roles in mRNA and tRNA quality processing and quality control, rRNA processing and ncRNA decay [2,3,5,6]. Xrn1 has several aliases [7], which reflect that it has been independently discovered on many occasions [3] with apparently multiple physiological and molecular roles. Its 5’→3’ exoribonuclease activity [8,9] is the reason for its name Xrn1 (exoribonuclease 1). Xrn1 5′→3′ exonucleolytic activity on mRNAs is central for the degradation of both correct and incorrect molecules, provided that they have a free 5’P end produced by either decapping or endonucleolytic cleavage [2,3]. It is a highly processive enzyme that can degrade untranslated mRNAs, but can also follow the last translating ribosome by cotranslationally degrading its mRNAs template [10,11]. Xrn1 has a paralog in most eukaryotes [12], called Xrn2/Rat1 in yeast [13], which is only nuclear, but can partially substitute Xrn1 when made cytoplasmic [14].

This large protein also performs other functions not directly related to its exoribonuclease activity. We have previously demonstrated that it can travel to the nucleus and act as a transcriptional activator [15] by modulating both transcription initiation and elongation [16]. Xrn1, therefore, shuttles between the cytoplasm and the nucleus to participate in both transcription and mRNA degradation by acting as a homeostatic factor that balances synthesis rates (SRs) and decay rates [16–18]. In the cytoplasm, Xrn1 also promotes the translation of a specific group of transcripts that encode membrane proteins. So, for this group of mRNAs, Xrn1 stimulates transcription, translation and decay [19].

The proper cytoplasmic function of Xrn1 involves its shuttling between the cytoplasm and the nucleus [17], which is made possible by the recently discovered existence of two nuclear localization sequences (NLSs) [20]. In this work, our goal was to find if the cotranslational mRNA decay of Xrn1 depends on its nuclear shuttling. For that purpose, we used a strain containing an Xrn1 version with the two mutated NLSs (Xrn1^ΔNLS1/2^), which renders it constitutively cytoplasmic [20]. Our results show that blocking nucleo-cytoplasmic shuttling impairs 5’→3’ mRNA decay along coding regions for all mRNAs and specifically in the 5’UTR for a large group of mRNAs, which we call KDIS (Kem1 5’-Decay Import Sensitive). Finally, from the results of this paper and the comparison made to the study of Xrn1 substitution for an only-cytoplasmic version of Rat1 published elsewhere [21], we conclude that although cotranslational mRNA decay occurs in the cytoplasm, shuttling to the nucleus is required for the full *in vivo* functions of Xrn1.

## Materials and methods

### Yeast strains and culture conditions

**Fig 1:**
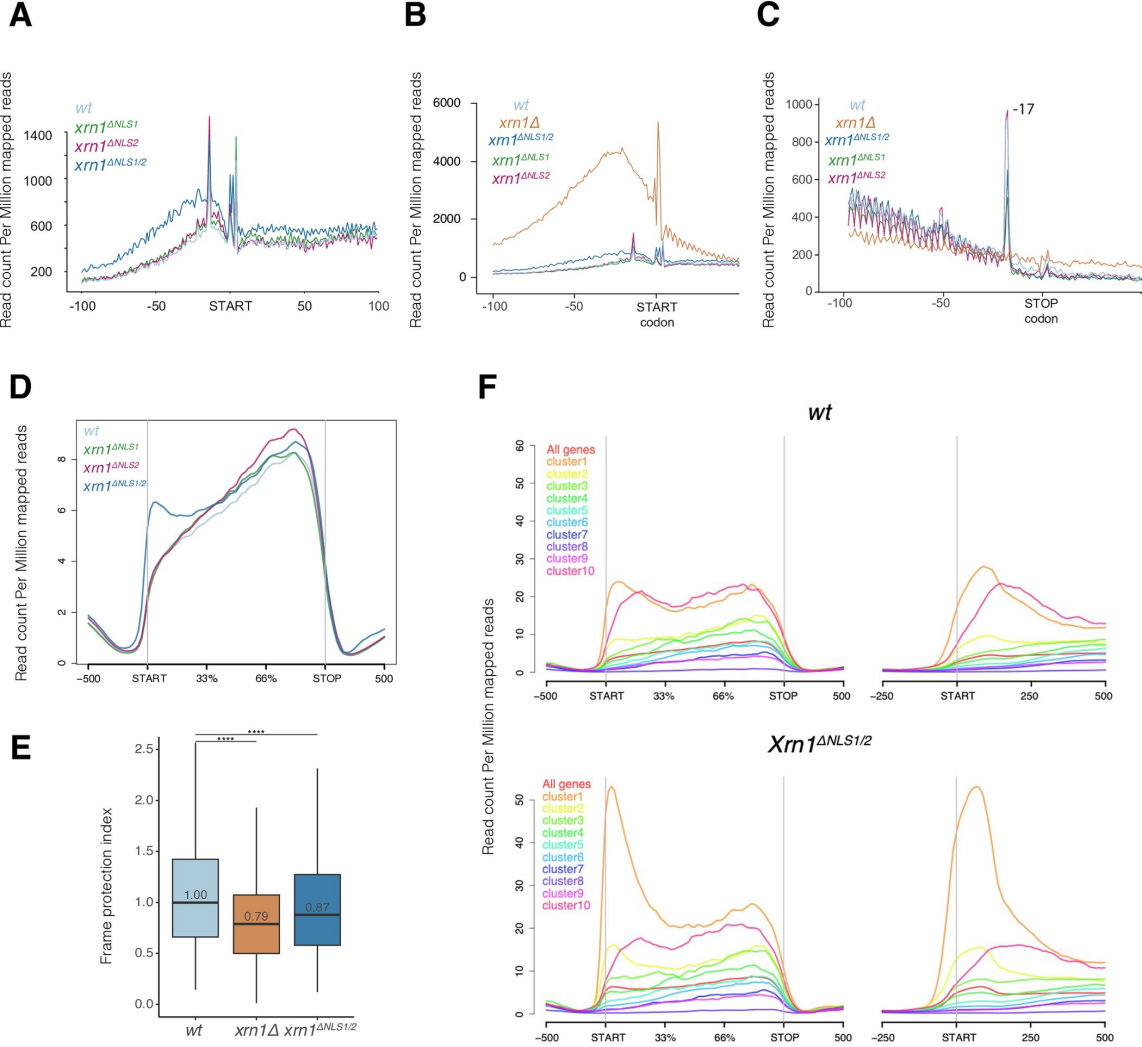
The only-cytoplasmic version of the Xrn1 protein (Xrn1^ΔNLS1/2^) partially restores the wild-type HT-5Pseq profile. A-C) The high-resolution metagene analysis for the HT-5Pseq read coverage in relation to the ORF start and stop codon for the wild-type (*XRN1*) with a version lacking NLS1 (*xrn1*^*ΔNLS1*^) or NLS2 (*xrn1*^*ΔNLS2*^), or both NLSs (*xrn1*^*ΔNLS1/2*^). In A and B the region around the start codon is shown at two Y scales. In A it can be seen the differences between the samples analyzed in this study. In B and C an *xrn1Δ* sample from another study [21] normalized for the total reads is added as a reference of a strain with no cytoplasmic 5’→3’ exoribonuclease activity. Note that the scales in both panels, B (around START codon) and C (around STOP codon), differ. Please note that the 3 bp periodicity seen in *xrn1Δ* is different (displaced -1nt) from the one observed in the wild-type cells. In *xrn1Δ*, the periodicity is likely caused by the activity of endonucleases in absence of cytoplasmic 5’-3’activity combined with the uneven distribution of nucleotides along the coding region. Thus, it does not inform regarding single-nucleotide ribosome positions as explained in reference [10]. In C the profile around the stop codon is shown. -17 marks the strong peak caused by the ribosome paused at the stop codon. D) The average metagene plot of the HT-5Pseq normalized counts over the coding sequence and the flanking regions of all the protein-coding genes for the HT-5Pseq samples. Note that the X scale in the coding region is given in relative distance units, whereas the upstream START and downstream STOP regions come in natural units. The genebody-spanning metagene plots were generated using the default curve smoothing implemented in *ngs*.*plot* with the whole sequencing read. This contrasts to the plots from panel A, generated using the *Fivepseq* package, where only the most 5’ nucleotide was employed. This explains the apparent different position of the 5’ peak in both panels. A version of this plot including the *xrn1Δ* sample from another study [21] is shown in S3 Fig. E) A comparative boxplot of the relative frame protection index (FPI) values between the WT and *xrn1*^*ΔNLS1/2*^. The *xrn1Δ* sample from another study [21] is used as a reference. **** = p < 0.0001 using a Wilcoxon test. F) The average metagene plots showing the HT-5Pseq coverage around the entire coding sequence (left) or only around the START codon for each 10 k-means cluster from the WT dataset (top) and for *xrn1*^*ΔNLS1/2*^ (bottom). Note that the X scale in the coding region is given in relative distance units, whereas the upstream START and downstream STOP regions come in natural units in the left panels, and the expanded scale in the right panels is expressed in only natural units. Also note that the Y scale in D & F panels are different from A-C panels as explained in M&M. Three biological replicates of the HT-5Pseq were done and averaged. In S4 Fig the individual tracks for wild-type and *xrn1*^*ΔNLS1/2*^ are shown.

For the experiments described in Fig 1, yeast cells were grown in YPD (1% yeast extract, 2% peptone, 2% glucose) at 30°C. Precultures were grown overnight in 250 mL flasks and shaken at 190 rpm. The next day, precultures were diluted to OD_600_ 0.05 and grown until an OD_600_ of ~0.5 was reached. Cells were recovered by centrifugation, flash-frozen in liquid nitrogen and stored until needed for RNA extraction. The yeast strains are listed in S1 Table.

For the experiments described in Fig 3C and 3D, yeast cells were grown in synthetic complete (SC), synthetic dropout (SD) or YPD medium at 30°C unless otherwise indicated. For harvesting optimally proliferating cells, strains were grown for at least seven generations in the logarithmic phase before harvesting. For the growth curve, cells were grown for seven generations to reach the mid-log phase. Subsequently, cells were diluted to 0.1 to 0.2 OD_600_, and growth was measured after the indicated times in a standard spectrophotometer. For the drop dilution test, cells were grown for seven generations to reach the mid-log phase, after which they were diluted to obtain 10^5^ cells/ml in each sample. Every sample was then serially diluted at the 1:10 ratio 4 times. Next 8 μl of each dilution were spotted onto a synthetic complete (SC) plate or a synthetic plate with glycerol as the sole carbon source. Plates were imaged at different time points to observe growth.

### Western analysis of Xrn1 variants expression levels

Western analysis of Xrn1 variants was performed as previously described [20] using cells expressing FLAG-tagged Xrn1, or its mutant derivatives. The membrane was incubated with anti-FLAG antibody and anti-ATP2 which was used as a loading control.

### Effect of S454P mutation on mRNA degradation and NMD

The strains carrying the wild-type (WT), mutated or deleted Xrn1 were transformed with the RPL30pG-containing plasmid [22]. Transformants were grown for seven generations to reach the mid-logarithmic phase, cells were harvested and RNA was extracted. For the mRNA degradation and the NMD experiments, RNA was respectively run on polyacrylamide gel and agarose gel, followed by northern blot analyses, performed as previously described [23].

### HT-5Pseq

RNA was purified by phenol extraction and the HT-5Pseq libraries were prepared as previously reported [24]. Briefly, 6 μg of DNA-free total RNA were directly ligated with the RNA/RNA oligo containing a unique molecular identifier (UMI) (RNA rP5_RND oligo). The ligated RNA was reverse-transcribed and primed with the Illumina PE2 compatible oligos containing random hexamers and oligo-dT The RNA in the RNA/DNA hybrid was depleted by sodium hydroxide with a 20-minute incubation at 65°C. The ribosomal RNAs were depleted using DSN (duplex-specific nuclease) with the mixture of the ribosomal DNA probes. Samples were amplified by PCR and sequenced in an Illumina NextSeq 500 instrument using 60 sequencing cycles for read 1 and 15 cycles for read 2.

The dataset for the HT-5Pseq data is available from the GEO database: GSE193992 (reviewer access code: *ahadwoocjrivtot*).

### Bioinformatics procedures

The HT-5Pseq reads were trimmed with a 3’-sequencing adapter using *cutadapt* V1.16 (http://gensoft.pasteur.fr/docs/cutadapt/1.6/index.html). The 8-nt random barcodes on the 5 ′ ends of reads were extracted and added to the header of the fastq file as the UMI employing UMI-tools. The 5‘P reads were mapped to *S*. *cerevisiae* (SGD R64-1-1) using *STAR 2*.*7*.*0* with parameter—*alignEndsType Extend5pOfRead1* to exclude soft-clipped bases on the 5′ end. After removing PCR duplications using UMI-tools, an analysis of the 5′ ends positions was performed with the *Fivepseq* package [25], including the relative distance to start and stop codons. In particular, the unique 5´ mRNA reads in the biological samples were summed and subsequently normalized to reads per million (rpm). The *Fivepseq* package also computes a frame protection index (FPI) for each gene (see the Materials & Methods section in ref. [10]). FPI evaluates the 5’P ends associated to the expected XRN1 trimming of ribosome occupied mRNAs vs 5’P ends occurring in other position independent of the ribosome occupancy. As such, it determines the extent of protection from 5’ to 3’ decay in the coding frame in relation to the other two frames. It measures the effectiveness of single-nucleotide coupling between nuclease activity and the ribosome position. FPI is computed with the formula: log_2_((2F_preferred_ frame)/(Ftotal—F_preferred_)). An FPI above than 0 indicates that the frame is preferred and provides a measure of distance between peaks and valleys for the 3-nt periodicity for each gene. The standalone normalized average density plots around the genomic features were calculated with the R and Python software *ngs*.*plot v2*.*61* [25] using indexed alignment files as inputs and the internal *SacCer3* database annotation as a reference. A particular feature of *ngs*.*plot* is that it calculates the “physical coverage” of reads around genomic features, instead of the traditional “read coverage". To achieve that, first the coverage vectors are normalized to be of equal length via a cubic spline fit, and then normalized again against the corresponding library size to be expressed as read counts per million mapped reads (RPMs). This approach is different to that of *fivepseq*, which result in conceptually similar metagene representations, but with different scales in the Y-axis [26]. The statistical robustness parameter, which filters out 5% of the positions with the most extreme (high and low) count values, was applied to all the calculations. A comparative gene ontology term enrichment analysis (GSEA) was performed with the *compareCluster* function from the R package *clusterProfiler* (https://guangchuangyu.github.io/software/clusterProfiler/) using the *org*.*Sc*.*sgd*.*db* annotation and the biological process ontology. GO term redundancy was reduced with the simplify function, which was implemented with the following parameters: cutoff = 0.5, by = "p.adjust", select_fun = min, measure = "Wang", semData = NULL. Having created the *compareCluster* object, the results were visualized with the *dotplot* function from the R package *enrichplot* (https://yulab-smu.top/biomedical-knowledge-mining-book/).

### Principal component analysis (PCA)

A PCA was performed with the relative values of the measured gene’s expression obtained by Genomic Run-On (synthesis rate, SR; mRNA amount, RA and mRNA stability, HL) in this study, and in previous ones [15,20,21] for several yeast mutants regarding their respective WT sample by performing the *PCA* function from the R [27] package *FactoMineR*. The PCA analysis allowed to summarize the information contained in the genomic datasets by focusing on whether they can discriminate the observations made by some previously obtained [15,20,21] features: cytoplasmic 5’→3’ exoribonuclease activity, formation of the decaysome complex, the cytoplasmic location of Xrn1 and the shuttling (nucleus-cytoplasm) of Xrn1. The genomic datasets employed in these analyses were from the Gene Expression Omnibus (GEO) with accession numbers: GSE29519, GSE158250.

## Results

### **Nucleo-cytoplasmic shuttling of Xrn1 is necessary for the proper 5’**→**3’ exonuclease decay of cytoplasmic mRNAs**

A recently discovered feature of Xrn1 is its capacity to shuttle between the nucleus and the cytoplasm. Its nuclear import is governed by two NLSs (NLS1 and NLS2), which are both necessary, i.e., the disruption of only one NLS compromises Xrn1 import, while the disruption of both NLSs abolishes it entirely. Following its import, Xrn1 stimulates transcription and then returns to the cytoplasm bound to mRNAs [20]. To explore whether cotranslational decay depends on the nucleo-cytoplasmic shuttling of Xrn1, we profiled the 5′phosphorylated mRNA degradation intermediates *via* high-throughput 5′P degradome RNA sequencing (HT-5Pseq) [24] after substituting the endogenous Xrn1 for a version lacking either one NLS (*xrn1*^*ΔNLS1*^, *xrn1*^*ΔNLS2*^) or both NLSs (*xrn1*^*ΔNLS1/2*^), in which the basic residues in both NLSs were substituted for alanines [20]. Given that HT-5PSeq is based on single-stranded RNA ligation, by targeting specifically the 5’P RNA molecules present in the cell, the bulk of the 5’capped mRNA molecules cannot be ligated and are, thus, omitted from the analysis. Therefore, the observed 5’ ends correspond to accumulated 5’P mRNA ends that underwent decapping (or endonucleolytic cleavage) and are still present in the cell. As those 5’P molecules are targets of Xnr1 mediated 5´-3´decay their accumulation indicates that they are protected from degradation by other factors (e.g. ribosomes) or that Xrn1 has not yet degraded them

The results showed that complete Xrn1 import inactivation, by the disruption of both NLSs (*xrn1*^*ΔNLS1/2*^*)*, resulted in an abnormal accumulation of HT-5Pseq reads mainly upstream of the AUG codon (Fig 1A, 1B and 1D). The disruption of a single NLS, which only lowered, but did not completely abolish the Xrn1 import [20], did not result in major alterations to the WT profile (see Fig 1A–1D). To show that the defect caused by the total absence of Xrn1 is much stronger than the one associated to its absence from the nucleus we compared the profiles with an *xrn1Δ* sample from another study [21]. The *xrn1Δ* sample showed a bulk of reads associated to decapped but not trimmed transcription start sites and a displaced 3-nt pattern likely associated to endonucleolytic cleavage in absence of cytoplasmic Xrn1 activity. Then we measured the ability of the exonuclease to follow the ribosome at a single-nucleotide resolution along the coding region, i.e., how good was the coupling between ribosome movement and exonuclease chasing. To do that, we used the previously defined “frame protection index” (FPI) [28] (see Fig 1E). Compared to the *xrn1Δ* strain FPI value (0.79), the *xrn1*^*ΔNLS1/2*^ strain recovered only 38% (FPI: 0.87) of the WT cotranslational decay capacity (set as 1.00). Therefore, Xrn1^ΔNLS1/2^ protein seems to be rather inefficient following the ribosome, i.e. blocking the Xrn1 nuclear import impairs cotranslational mRNA decay dynamics in coding regions. The observed changes were not due to an alteration of the protein expression level Xrn1^ΔNLS1/2^, as can be seen in S1 Fig.

Next, we wondered whether the cotranslational degradation defects observed in the average metagene profiles would affect the whole yeast transcriptome, or if they would impact only specific gene groups. Thus we divided the whole transcriptome dataset (5100 genes having transcription start and termination sites annotations) into 10 equal sized clusters according to their *xrn1*^*ΔNLS1/2*^/WT ratio in the 200 bp region around the START codon from the highest (cluster #1) to the lowest (#10). Then we represented the average profiles of each cluster as depicted in Fig 1D. We found a group of 1020 mRNAs (clusters 1 & 2), which are exceptionally sensitive to the disruption of NLS1/2. They displayed an abnormal accumulation of undegraded transcripts (in Fig 1F, lower panel). Interestingly, these clusters also had a higher signal along the whole transcribed region in the wild type (Fig 1F, upper panel, compare clusters #1 and 10), albeit much lower in the 5’ region than in the *xrn1*^*ΔNLS1/2*^ mutant. The mRNAs that underwent cotranslational degradation that depends on Xrn1 import to the nucleus were named “Kem1 5’-Decay Import Sensitive (KDIS)”. The other genes corresponding to clusters 3–10 (4080 genes: non KDIS) showed similar profiles in both the WT and *xrn1*^*ΔNLS1/2*^ strains (Fig 1F, compare both panels). This finding indicates that the alteration of the HT-5Pseq global decay profile (Fig 1D) was due mostly to the contribution of KDIS genes. Cluster #10 (the pink line in Fig 1F) contained a group of genes showing a non KDIS profile, but with a similar HT-5Pseq signal level to KDIS along transcribed regions. We used them as a comparison group in some of the following studies.

To rule out that the KDIS-specific profile (see Fig 1F) was caused by the indirect effect of Xrn1-nuclear depletion on non coding RNAs (ncRNA) levels, we analyzed if KDIS genes displayed any overlap with XUTs (Xrn1-sensitive Untranslated Transcripts) [29]. XUTs are a class of regulatory long ncRNAs targeted by cytoplasmic Xrn1. We found that only 24 of the 1020 KDIS genes had overlapping-sense XUTs in the 0.5 Kb region upstream of the TSS (p-value = 0.17, not shown). Therefore, a possible increase in the XUT levels in *xrn1*^*ΔNLS1/2*^ strain cannot explain the increase in 5’P seen in that region of KDIS genes. Moreover, to discard the potential existence of unannotated short cryptic XUTs overlapping the 5’region of canonical mRNAs in the same strand, we re-analyzed previously published 3’polyA RNA sequencing WT and *xrn1Δ* samples [30]. We did not find any clear difference between the WT and *xrn1Δ* strains, which suggests that defects in the XRN1 function do not lead to overlapping XUTs to appear that could explain the observed KDIS phenotype (S2A Fig). We conclude that the accumulation of 5’P molecules in the 5’region of KDIS genes is not caused by an increase in non coding sense overlapping transcripts because general Xrn1 exonuclease activity is lacking. So, the increase in the 5P degradation intermediates in the 5’region of KDIS should be a direct consequence of the inability of *xrn1*^*ΔNLS1/2*^ to localize in the nucleus. The conclusion on the 5’-decay defect in KDIS mRNAs is supported by the fact that they have slightly, but higher, mRNA level than the rest of mRNAs in the *xrn1*^*ΔNLS1/2*^ strain when comparing with the wild type using data from ref. [20] (S2B Fig).

**Fig 2:**
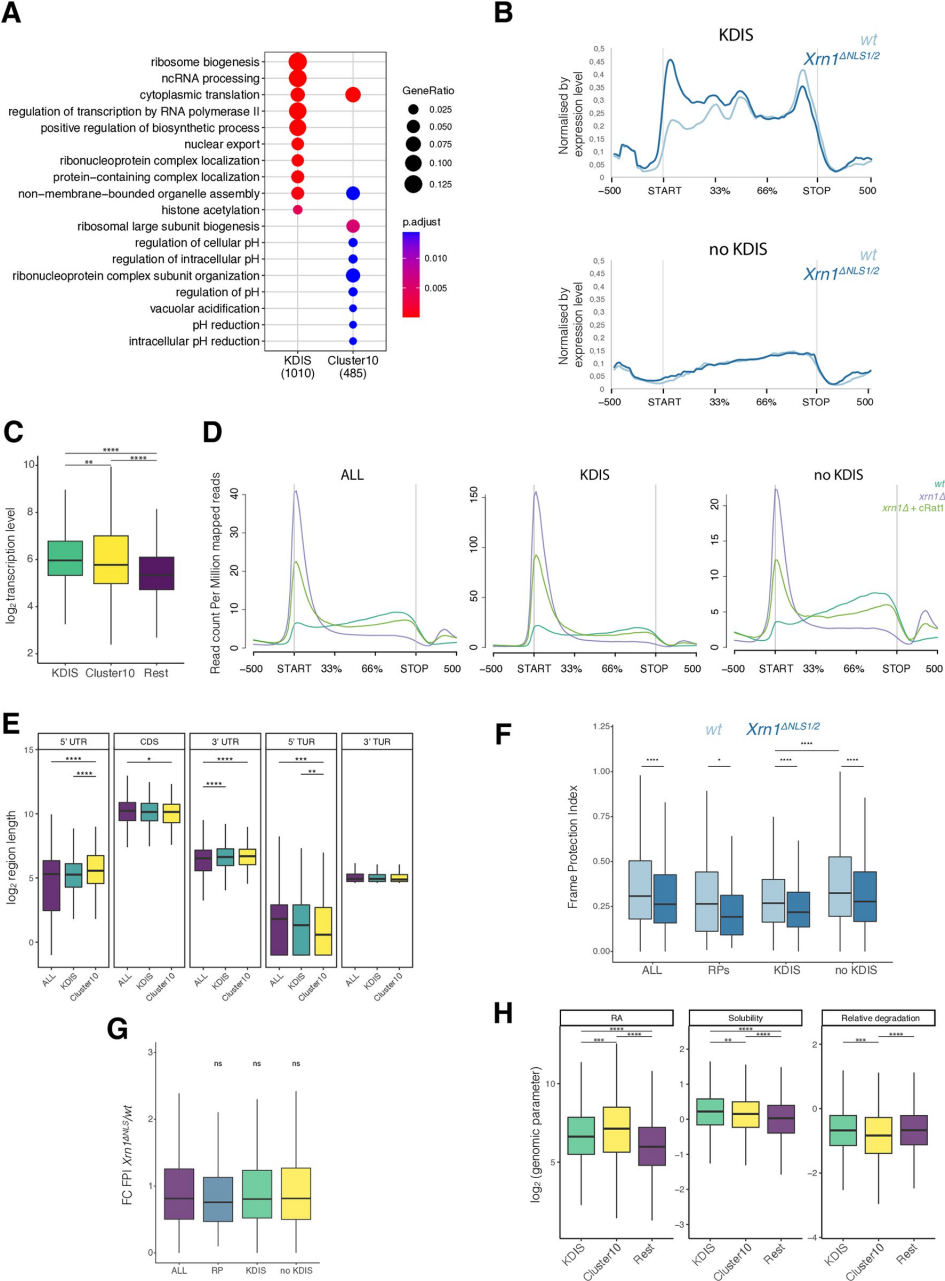
Characterization of the Kem1 5’-Decay Import-Sensitive (KDIS) genes. A) Mapping of the GO terms to KDIS and Cluster #10 genes. Only the top 10 enriched GO terms are shown, according to the adjusted p-value using a Benjamini-Hochberg (BH) test. Note the enrichment in GO terms related to cytoplasmic translation in both groups. B) The average metagene plots of KDIS and non KDIS after normalizing by their mRNA level values [20]. C) Comparison of the transcription levels of KDIS (n = 1020), cluster #10 (n = 510) and the other (non KDIS, n = 5134) genes. We used the BioGRO values from a wild-type (WT) strain [31] as a measure of the nascent transcription rates. D) The average metagene profiles of all the protein-coding genes (n = 6664), KDIS (n = 1020) and non KDIS (including cluster #10, n = 5644) for the cRat1 strain described in [21] compared to the WT and *xrn1Δ*. E) Boxplots showing the length of 5’UTR, CDS and 3’UTR and the 5’TUR and 3’TUR (from ref. [32]) of KDIS and cluster #10 genes. F) Comparison of the FPI values of the different groups of genes in *xrn1*^*ΔNLS1/2*^ *vs*. the WT. G) Comparison of the fold change of the FPI values in the *xrn1*^*ΔNLS1/2*^/WT for different groups of genes. All the compared groups display a similar drop in the FPI in *xrn1*^*ΔNLS1/2*^. H) Boxplots showing mRNA abundance (RA), abundance in the soluble fraction and relative 5’-degradation (from ref. [33]) of KDIS and cluster #10 genes. The significance of the median comparisons in panels C, E-H, was estimated using a Wilcoxon test: ns = p>0.05; * = p< 0.05; ** = p< 0.01; *** = p< 0.001; **** = p< 0.0001.

We next looked for the functional features of KDIS genes. We have previously found that a class of yeast mRNAs, enriched in the genes encoding membrane proteins, is dependent on Xrn1 for their translation initiation [19]. KDIS genes show no significant overlapping with either those genes or the genes that depend more on Xrn1 for transcription activation and mRNA decay, a group that we called synthegradon (S2C Fig) [18]. We also performed gene ontology term enrichment by performing a Gene Set Enrichment Analysis (GSEA) on the lists of the KDIS and cluster #10 genes. The KDIS and cluster #10 gene groups were both enriched in terms related to the cytoplasmic translation GO category, whereas KDIS were also enriched in Ribosome Biogenesis (RiBi) and cluster #10 in the categories related to cellular pH regulation (Fig 2A). We suspected that GO categories related to transcription factors were overrepresented in the KDIS (not shown). Therefore, we decide to pursue a manual curation of the KDIS genes. We found a statistically significant enrichment of KDIS in genes which code for chromatin-bound proteins, such as transcription factors, histones, histone-modifying enzymes and chromatin-remodeling complexes (hypergeometric test p-value 4.0 10^−7^). We also found that both KDIS and cluster #10 were more transcribed on average than the rest of the genome (Fig 2C), although this increased mRNA synthesis rate was not the cause of the observed molecular phenotype because normalization by the mRNA abundance levels did not significantly change the difference seen at the 5’ region between KDIS and non KDIS in the average metagene profile (Fig 2B). We compared this result to that of a yeast strain, where we substituted Xrn1 for a cytoplasmic version of Rat1 5’→3’exonuclease (cRat1), published elsewhere [21]. We found that KDIS lacked a distinct HT-5Pseq profile in either the *xrn1Δ* or *xrn1Δ*+cRat1 mutants (Fig 2D) compared to the other genes. This further reinforces that KDIS behavior is specific for the *xrn1*^*ΔNLS1/2*^ strain as the substitution of Xrn1 by cRat1 did not modify their phenotype.

The KDIS 3’UTRs (but not their 5’UTRs and coding regions) were longer than the average of the genome (Fig 2E). The cluster #10 genes have longer 5’UTR and 3’UTRs. Further inspection revealed that, similarly to the ribosomal protein (RP) genes, KDIS had lower FPIs than non KDIS in a WT strain (Fig 2F, non KDIS *vs*. RPs and KDIS). Interestingly, the FPIs for RPs, KDIS and non KDIS decreased to a similar extent in the *xrn1*^*ΔNLS1/2*^ mutant in relation to the WT strain (Fig 2F–2G). This suggests that, apart from the KDIS-specific 5’ accumulation of the HT-5Pseq reads (Fig 1B), blocking the Xrn1 nuclear import has a global, but not specific, effect on cotranslational mRNA decay dynamics in coding regions. We also analyzed the length of the terminal unstructured 5’ and 3’ regions (called 5’TUR & 3’TUR in reference [32]) needed for mRNA decay (Fig 2E). Our results indicated that neither the 5’TUR nor 3’TUR of KDIS significantly differed from the other mRNAs. However, the non KDIS genes included in cluster #10 tended to have a significantly lower 5’TUR value.

Further analyses of KDIS genes showed that their mRNAs tended to be enriched in the “soluble” fraction of the transcriptome (Fig 2H center). This finding was in accordance with the definition from [33], which classifies mRNAs according to their extractability from yeast cells. However, they were not significantly more 5’-degraded than the bulk of the genes (KDIS vs. Rest, Fig 2H right). Interestingly, the cluster #10 genes, which are the most abundant mRNAs (Fig 2H left) and enriched in the soluble fraction (Fig 2H center), were less 5’-degraded that the rest (Fig 2H right).

Therefore, we can conclude that, although KDIS have a clearly distinct degradation profile (Fig 1D), they did not show a clear distinctive features pattern in terms of mRNA abundance, GO terms, the 5’UTR secondary structure, length or extractability. Hence KDIS seems to be a feature that is related to the function(s) of Xrn1 in the nucleus.

### Principal component analysis of the yeast strains with different types of 5’→3’ exonuclease

We next explored the potential phenotypic effects of the *ΔNLS1/2* mutation beyond its impact on the cotranslational degradation profile. Xrn1’s shuttling behavior concept has recently been recognized for its significant role in mRNA decay [20]. To explore the physiological consequences of mutations in Xrn1’s NLSs, we ran a principal component analysis (PCA) on the genomic datasets from several yeast strains. Each one exhibited different characteristics for their major nuclear/cytoplasmic localization, cytoplasmic 5’→3’ exoribonuclease activity and shuttling behavior.

To assess how these several features of the different yeast strains having mutated versions of Xrn1, and another strain expressing the cytoplasmic version of the 5’→3’ exoribonuclease paralog cRat1, influenced their transcriptomic characteristics, we performed a PCA analysis on the genomic datasets obtained through genomic run-on (GRO) experiments. These datasets encompassed the: (i) previously published data on the synthesis rates (SR), mRNA amounts (RA) and half-lives (HL) of both *xrn1Δ* and the catalytically inactive *xrn1*^*D208A*^ mutant cells ([15]; dataset #1); (ii) data from a previous study of *xrn1*^*ΔNLSs*^ mutants and the *xrn1*^*S454P*^ mutant, which exhibited defective mRNA binding ([20]; dataset #2); (iii) data from studying WT and *xrn1Δ* cRat1-transformed strains ([21]; dataset #3). For the PCA analysis, we utilized the phenotypic features collected from these prior publications [15,20,21], including the: i) existence of cytoplasmic 5’→3’ exoribonuclease activity; ii) formation of the decaysome complex; iii) cytoplasmic localization of the Xrn1 protein; iv) shuttling capacity of 5’→3’ exonuclease.

**Fig 3:**
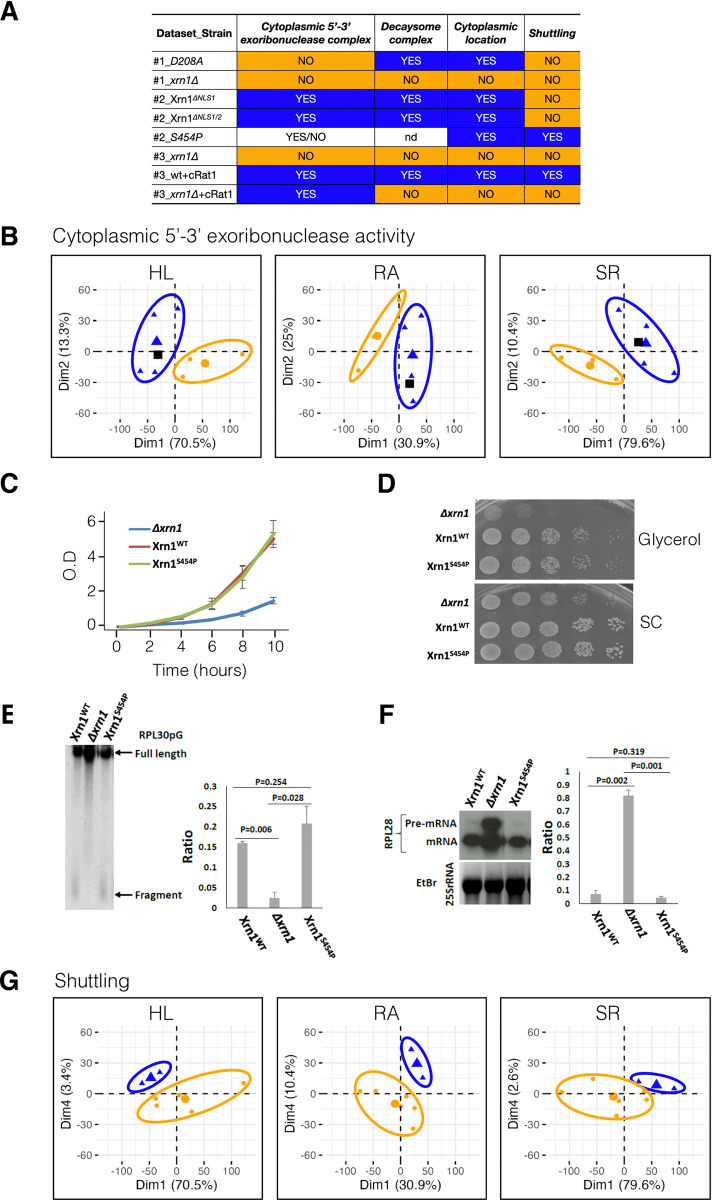
Principal component analysis (PCA) of the transcriptional genomic data of several yeast strains with different 5→3’exonuclease activity. We used the genome-wide GRO data described in the main text from previously published three experiments: (ref. [15]: Dataset #1, ref. [20]: Dataset #2 and ref. [21] (dataset #3). A) The table shows the known features (from refs. [15,20,21]) that characterize the studied strains marked by different colors (YES in blue, NO in orange) corresponding to the dot colors in parts B and E. Note that *xrn1*^*ΔNLS1*^ is classified as deficient in shuttling because it has only a very minor capacity of nuclear Xrn1 import (see [20]). B) How Dimensions 1 and 2 in the PCA analysis separate the individuals with YES and NO in the “5’→3’decay” feature for the mRNA half-life (HL), the mRNA concentration (RA) and the mRNA synthesis rate (SR) is depicted. Blue triangles correspond to the strain samples which have the 5’→3’decay capacity (YES in panel A); orange dots depict the samples without it (NO in part A). The black square corresponds to the undetermined *S454P* sample. Big triangles and dots represent the centroid (mean) of the group highlighted by an oval. C) Growth rate shown as O.D. *vs* time of culture for the wild-type (WT), *xrn1Δ* and *xrn1*^*S454P*^ strains used for the *S454P* mutation test. D) Growth of same strains in media with glycerol (as the sole carbon source) and in synthetic complete (SC) medium. E) Effect of *S454P* mutation on degradation, measured by Northern blotting. Degradation of RPL30pG [33] in the WT, the *xrn1*^*S454P*^ mutant and *xrn1Δ*; degradation of RPL30pG leaves a Poly-G tract as a degradation fragment. The ratio of the fragment to the full-length RPL30pG is presented here. The results represent the averages of two replicates (±SD). F) Effect of the *S454P* mutation on NMD, measured by Northern blotting. A representative example is shown. In this image a few irrelevant lanes were removed, but otherwise the contrast and intensity were not changed. The ratio of mRNA to the pre-mRNA of RPL28 is presented in the right graph. The results represent the averages of two replicates (±SD). G) Dimensions 1 and 4 separate the groups for the “shuttling” feature. Symbols as in part B). nd, not determined. All the other binary combinations for dimensions 1 to 4 showed no significant clustering for the analyzed features (not shown).

We performed PCAs for all the possible combinations of four different dimensions for each mutant strain using relative values (for HL, SR, RA) compared to the WT strain across all three experiment datasets. Fig 3A is a binary matrix that summarizes the strain features used in PCAs, which indicates the presence (YES, blue-highlighted) or absence (NO, orange-highlighted) of these features for different strains. In most cases, our analysis did not reveal any significant groupings of strains based on the analyzed features (not shown). However, the PCA was able to distinguish the ability to perform cytoplasmic 5’→3’ decay. As shown in Fig 3B, dimension 1 (along with dimension 2) effectively discriminates between strains with the presence (depicted as blue triangles) and absence (represented by orange dots) of cytoplasmic 5’→3’ exonuclease activity, which is provided by the WT (*XRN1*), *xrn1*^*ΔNLS1/2*^ or cRat1 strains. Our finding reinforces the notion that cRat1 can effectively complement a primary physiological function of Xrn1, i.e., cytoplasmic 5’→3’ exonuclease activity [21]. The black square, representing the *xrn1*^*S454P*^ strain, which lacks a clear classification in the original study [20], is grouped with the positive samples in this analysis. What this scenario suggests is that this Xrn1 mutant possesses 5’→3’ exonuclease activity.

To validate this last prediction and to assess the reliability of the PCA for predicting features related to Xrn1, we conducted an experimental study. In this experiment, we introduced a modification to the *xrn1*^*S454P*^ strain by transforming it with a version of the RPL30 gene, named RPL30pG, which contains a polyG tract at the 3’ end of its mRNA [22]. Notably, this modified strain exhibited similar growth characteristics to the *xrn1*^*S454P*^ strain, which is, in turn, similar to that of the wild type [20] (Fig 3C and 3D). The presence of the polyG tract serves to block 5’→3’ exoribonuclease activity progression. Hence the appearance of a fragment spanning from the polyG tract to the mRNA’s 3’ end during northern blot hybridization (using oligo dC as probe) indicates the existence of the catalytic 5’→3’ exonuclease activity responsible for degrading RPL30pG mRNA. As depicted in Fig 3E, the *xrn1Δ* strain lacks 5’→3’ exoribonuclease activity, while both the WT and *xrn1*^*S454P*^ strains possess this activity. Furthermore, the *xrn1Δ* strain exhibits deficiency on the nonsense-mediated mRNA decay (NMD) pathway for degrading intron-containing mRNAs, such as RPL28. Fig 3F evidences that both the WT and *xrn1*^*S454P*^ strains can effectively degrade RPL28 intron-containing mRNAs. These results serve to validate the effectiveness of our PCA approach in gaining biologically relevant insights from our genomic datasets.

In addition to the cytoplasmic 5’→3’ exoribonuclease feature, the PCA found for the shuttling feature (Fig 2G), dimension 1 (in combination with dimension 4) that discriminates between positive (blue triangles) and negative (orange dots) samples by indicating a minor, but significant, contribution of the shuttling between the nucleus and the cytoplasm to the physiological function of Xrn1. Interestingly, we have classified *xrn1*^*ΔNLS1*^ as shuttling-deficient because, previously, we found that the NLS1 mutation abolishes most (but not all) nuclear import capacity of Xrn1 [20]. This classification is validated by PCA, despite 5’-P-seq results (Fig 1] that showed that the presence of a small amount of nuclear Xrn1 is sufficient to abolish Xrn1 shuttling-dependent phenotypes. Thus, the PCA supports the notion that the Xrn1 shuttling feature is a significant property that significantly affects transcription process in yeast.

## Discussion

Xrn1 is a highly conserved, and exceptionally large, protein (175 kDa) with multiple biological functions in many eukaryotes. Many of these functions can be explained by its enzymatic 5’→3’ exonuclease activity acting on many different RNA types. All their RNA substrates require a single-stranded 5’phosphate end with a length of at least 4 nt [3]. This multifunctional protein is abundant and mainly cytoplasmic, but also plays many roles in the nucleus. To perform nuclear functions, it shuttles between the cytoplasm and the nucleus [15,17,18] using two distinct NLSs [20]. Its export to the cytoplasm has been proposed to be mediated by its ability to bind or “imprint” mRNAs [20,34]. Proper Xrn1 nucleo-cytoplasmic shuttling and *mRNA imprinting* have been shown to be important for the regulation of mRNAs synthesis and decay, a feature that is critical for responding to changes in the environment [20]. In this work, we studied how blocking shuttling impacts the various functions of Xrn1, including its ability to degrade ribosome-bound mRNAs during their last translation round in the cytoplasm.

Regarding the cotranslational decay phenotype, the global effect of Xrn1 import on the FPI, and the comparative analysis of *xrn1*^*ΔNLS1/2*^ (Fig 1E) with the WT, suggests that cytoplasmic Xrn1 is rather inefficient (38% increase in *xrn1*^*ΔNLS1/2*^ regarding *xrn1Δ*, taken as a background noise reference) to perform cotranslational 5’→3’ mRNA decay along coding regions. The Xrn1 shuttling is, therefore, necessary for wild type cotranslational decay in coding regions following the last translating ribosome. Although an indirect effect (via changes in some mRNA levels) cannot be ruled out, we propose that Xrn1 shuttling has direct effects on the observed phenotypes (see below).

Additionally, *xrn1*^*ΔNLS1/2*^ is unable to degrade 5’UTRs of a particular group of mRNAs, called KDIS (Figs 1D, 1F and S3). This effect is specific of this strain given that cRat1 cells lack the difference in the HT-5Pseq profile between KDIS and non KDIS genes (compare KDIS to no KDIS in Fig 2D), which suggests that only Xrn1, particularly its shuttling feature, is necessary for the initiation of cotranslational decay of KDIS mRNAs. As, HT-5PSeq measures only 5’P mRNA degradation intermediates, our results suggest that KDIS 5’UTRs can be decapped but not efficiently degraded. This idea is supported by the PCA results because we found that the shuttling feature was a common property for discriminating the transcription and mRNA decay of several mutant yeast strains (Fig 3G). Hence this scenario reinforces the notion that nucleo-cytoplasmic shuttling is an essential part of Xrn1 biology [20]. Although we do not know the molecular mechanism by which the nuclear import of Xrn1 affects the cotranslational decay of KDIS mRNAs, we attempted to discard indirect effects due to altered XRN1 activity. We ruled out the possibility that the accumulation of 5’P reads in the 5’regions of KDIS (Fig 1F) could be due to overlapping ncRNAs emerging as a consequence of lesser XRN1 activity. Therefore, the depletion of XRN1 does not lead to the accumulation of XUTs [28] in either proximity or overlapping the identified KDIS (see S2A Fig). Furthermore, the KDIS phenotype is based on the differential relative accumulation of 5’P degradation intermediates along the gene. Thus, changes in gene expression (i.e., mRNA abundance) cannot explain the observed phenotype. Interestingly, KDIS genes have no overlapping with the previously defined 2401 KIS (Kem1 import-sensitive) genes, which were defective in both synthesis and decay rates (≥ 2-fold) when blocking Xrn1 import in a *xrn1*^*ΔNLS1/2*^ mutant [20]. Currently, we have no explanation to this finding and it would require further research. However, this suggests that Xrm1 shuttling affects multiple mRNA synthesis and decay pathways.

The structural and functional analysis of KDIS genes shows that they are a special group that does not overlap other genes regulated by Xrn1, such as those we identified as being translationally activated by cytoplasmic Xrn1 [19]. KDIS genes, characterized by high transcription and mRNA levels, and with many transcripts involved in translation-related processes, do not significantly overlap synthegradon genes (S2B Fig), which are also enriched in the GO categories related to translation [18]. KDIS are also enriched in chromatin-related proteins. We hypothesize that KDIS mRNAs could be imprinted by Xrn1 or by other factor in a Xrn1-dependent way. In that case the absence of nuclear Xrn1 would avoid imprinting and cause, potentially, the decay defects of KDIS genes.

According to a recently published work [33], KDIS mRNAs are enriched in a “soluble” fraction purified from yeast cells after soft RNA extraction. What that study revealed was that those “soluble” mRNAs were less prone to cotranslational decay (lesser abundance of the 5’P degradation intermediates *vs*. mature mRNAs) than in the total RNA pool (including the “insoluble” pool). The total RNA pool is obtained by conventional hot-phenol extraction and would include membrane-associated mRNA, condensates and other aggregates. Insoluble mRNAs would be more cotranslationally decayed (higher relative abundance of 5’P degradation intermediates *vs*. mature molecules). These hypotheses fall in line with our characterization of KDIS genes, which behave as cotranslationally decay-resistant in the *xrn1*^*ΔNLS1/2*^ mutant and mostly come in the “soluble” fraction. However, the mRNAs included in cluster #10 are also enriched not only in the translation-related GO categories, but also in the “soluble” fraction (Fig 2H), but are less degradable than both KDIS and the rest of the transcriptome according to reference [33] definition (Fig 2H). This suggests that the soluble fraction contains not only cotranslational resistant mRNAs (KDIS), but also some other abundant mRNAs that are actively cotranslationally degraded apart from those associated with membranes, as described by Allen et al. [33].

Apart from the effects on cotranslational mRNA decay our previous studies showed that the most relevant phenotypic defects caused by lack of cytoplasmic 5’→3’ exoribonuclease activity can be solved mostly by a cytoplasmic version of either Rat1 or Xrn1 enzymes. The *xrn1*^*ΔNLS1/2*^ strain has no defect on the growth rate in rich media [20] whereas the cRat1 strain has only a minor defect (22% slower than the WT, see [21]). Both strains are similar in cell size to a WT strain [20,21] and in the global transcriptional pattern (as shown by our PCA study). These results show that cytoplasmic 5’→3’ is the physiological most relevant function of Xrn1 under non-stressful conditions. However, the PCA study also shows that the shuttling capacity of the natural Xrn1 protein is a relevant feature needed for its full *in vivo* function. In fact, in previous studies, we have observed a 37% decrease in the mRNA SRs in relation to the wild type in an *xrn1Δ* strain expressing a cytoplasmic version of Rat1 (cRat1) [21]. In another study, we have also shown that the *xrn1*^*ΔNLS1/2*^ mutant has 53% lower average SRs than those in the WT strain [20]. Note that both strains, cRat1 and *xrn1*^*ΔNLS1/2*^ lack the nuclear location of Xrn1, but differ in terms of the presence in the cytoplasm of the natural Xrn1, instead of non natural Rat1 5’→3’ exoribonuclease activity, in the second strain. We have previously demonstrated that Xrn1 acts as a master regulator for importing several components of the decaysome complex and travels with them from the cytoplasm to the nucleus [15,20]. We propose that if Xrn1 is retained in the cytoplasm (as in *xrn1*^*ΔNLS1/2*^ cells), the whole decaysome would also be retained in the cytoplasm and the negative effect on SRs would be enhanced in relation to the cRat1 strain. This decaysome complex retention effect has been previously reported for the *xrn1*^*D208A*^ enzyme dead mutant, which is also unable to travel to the nucleus [15].

All these results lead us to propose that Xrn1 shuttling between the cytoplasm and the nucleus shapes the yeast transcriptome. This is done in such a way that it affects global cell physiology, mainly during stress responses [20]. Xrn1 shuttling is not equally important for the cotranslational decay of all mRNAs because some, namely those we call KDIS, need shuttling for proper cotranslational decay, whereas the rest of the transcriptome does not. The fact that not only Xrn1 shuttling is important for rapid transcriptional responses to changes in the environment [20], but also that KDIS genes need it for cotranslational decay, may indicate that rapid response and cotranslational decay are functionally related. This suggestion is supported by the fact that KDIS genes are enriched in GO categories, such as translation and RiBi, which are closely related to yeast growth.

## Supporting information

S1 Fig Western analysis of Xrn1 variants. A) Equal amount of whole-cell extracts, taken from optimally proliferating cells expressing FLAG-tagged Xrn1, or its indicated mutant derivatives (see S1 Table), were analyzed by western blot. Membrane was incubated with anti-FLAG antibody and with anti-ATP2 that was used as a loading control. B) Quantification of immunoblots: Images were acquired using ImageQuant and quantification of western blot bands were done using Image J software. Signal of Xrn1 was normalized to that of Atp2. Three biologically independent samples were averaged except for S454P and NLS1 where only two were considered. Error bars represent standard deviation (SD). No significant differences were observed with regard the wild type sample using Student’s unpaired T-test for any of the Xrn1 variants.
(PDF)

S2 Fig Characterization of KDIS genes. A) Heat map displaying the measured poly(A) site reads for the wild-type (WT) and a *xrn1Δ* strain for KDIS and non KDIS genes. As expected, the bulk of the poly(A) site reads occur downstream of the stop codon, and a small fraction occurs prior to the start site (as expected from those originating from the upstream genes in tandem). KDIS genes do not display an increased number of poly(A) reads downstream of the start codon in the *xrn1Δ* strain. This suggests that XRN1 depletion does not lead to the accumulation of overlapping cryptic transcripts over the 5’region of KDIS. Plot generated from GSE40110 and GSE158548 using *DeepTools* [35]. Three individual replicates of WT and two of *xrn1Δ* strains are shown. B) Boxplots showing mRNA levels of KDIS vs. the rest of the genes (data from ref. [20]). The significance of the median comparisons was estimated using a Wilcoxon test: * = p< 0.05. C) Statistical study of KDIS genes. Venn diagrams show that KDIS genes do not significantly overlap those genes that depend more on Xrn1 for transcription activation and mRNA decay (*synthegradon*, Ref. [18]) and those genes whose mRNAs depend on Xrn1 for translation activation [19]. Statistical significance for every pair-wise comparison is indicated.
(PDF)

S3 Fig Comparative metagene analysis for the HT-5Pseq in ΔNLS strains with *xrn1Δ* mutant. The same plot shown in Fig 1D showing the coverage in relation to the ORF start and stop codon for the wild-type (*XRN1*) with a version lacking NLS1 (*xrn1*^*ΔNLS1*^) or NLS2 (*xrn1*^*ΔNLS2*^), or both NLSs (*xrn1*^*ΔNLS1/2*^) is shown together an *xrn1Δ* sample from another study [21] normalized for the total reads added as a reference of a strain with no cytoplasmic 5’→3’ exoribonuclease activity. Note that the Y scales of this figure and Fig 1D are different.
(PDF)

S4 Fig Individual profiles comparison for the three replicates of wild type and *xrn1*^*ΔNLS1/2*^ samples. High-resolution metagene analysis for the HT-5Pseq read coverage in individual three replicates of the wild-type (*XRN1*) or lacking both NLSs (*xrn1*^*ΔNLS1/2*^). The averaged plots are shown in Fig 1A–1C. Note the similarity of the replicates for each sample and the differences between wild type and *xrn1*^*ΔNLS1/2*^.
(PDF)

S1 Table List of the yeast strains used in this work. (XLSX)
