# Supplementary figures and images for: Proper 5’-3’ cotranslational mRNA decay in yeast requires import of Xrn1 to the nucleus
Source: PLoS One. 2025 Jan 22;20(1):e0308195. doi: 10.1371/journal.pone.0308195 (PMC11753706; doi:10.1371/journal.pone.0308195)

**A**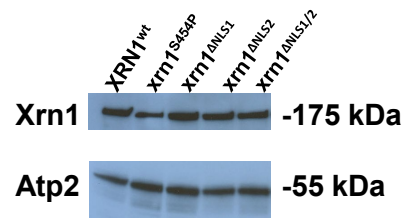**B**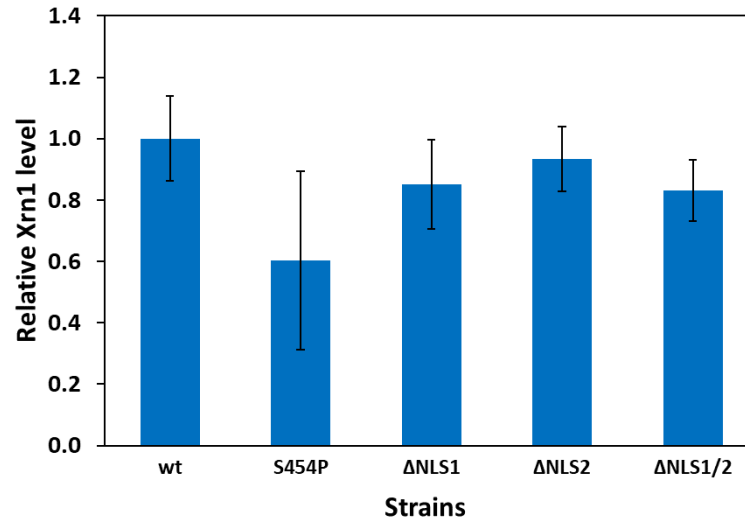**Figure S1**

Supplement: S1 Fig — A) Equal amount of whole-cell extracts, taken from optimally proliferating cells expressing FLAG-tagged Xrn1, or its indicated mutant derivatives (see S1 Table), were analyzed by western blot. Membrane was incubated with anti-FLAG antibody and with anti-ATP2 that was used as a loading control. B) Quantification of immunoblots: Images were acquired using ImageQuant and quantification of western blot bands were done using Image J software. Signal of Xrn1 was normalized to that of Atp2. Three biologically independent samples were averaged except for S454P and NLS1 where only two were considered. Error bars represent standard deviation (SD). No significant differences were observed with regard the wild type sample using Student’s unpaired T-test for any of the Xrn1 variants. (PDF) [file pone.0308195.s001.pdf]

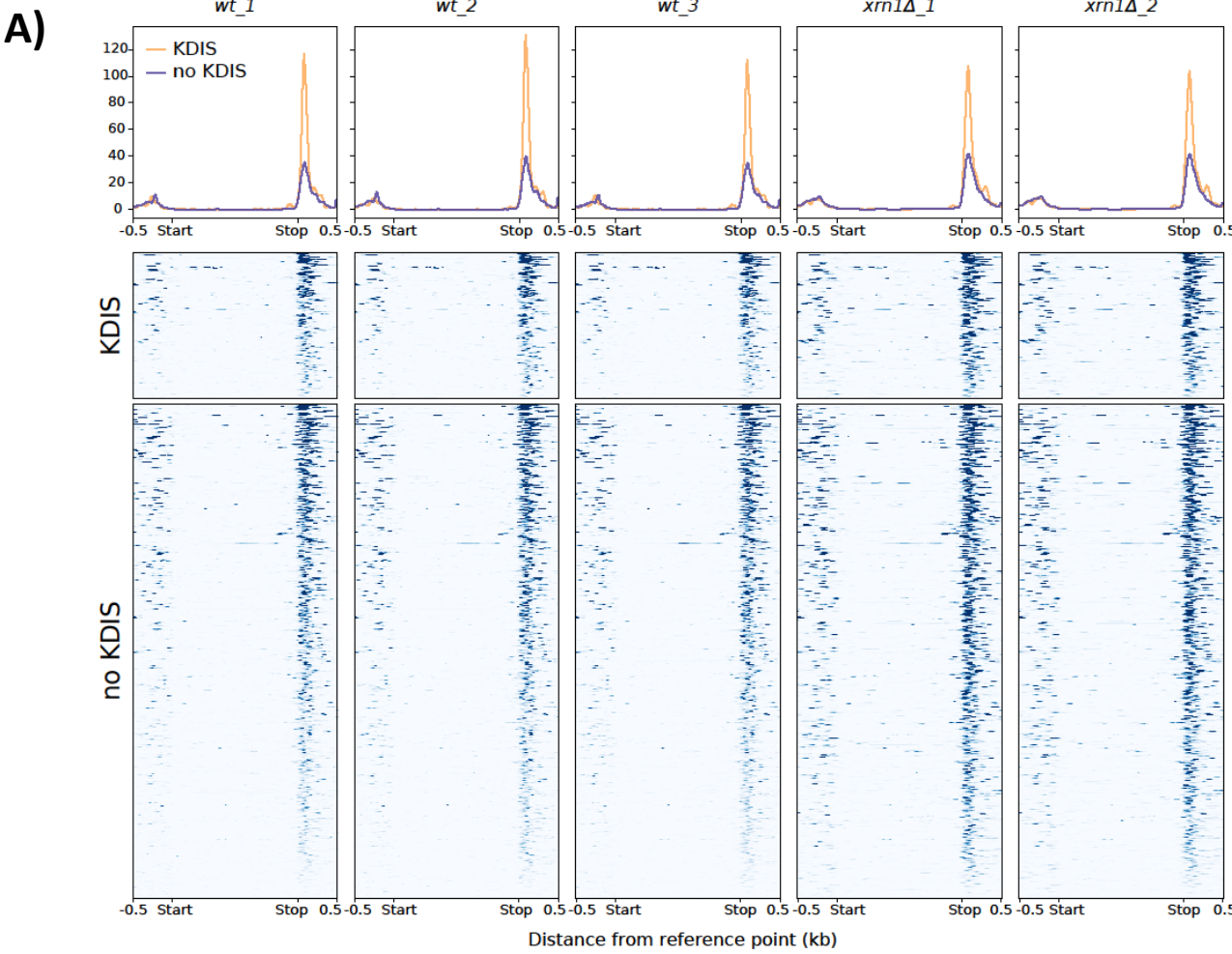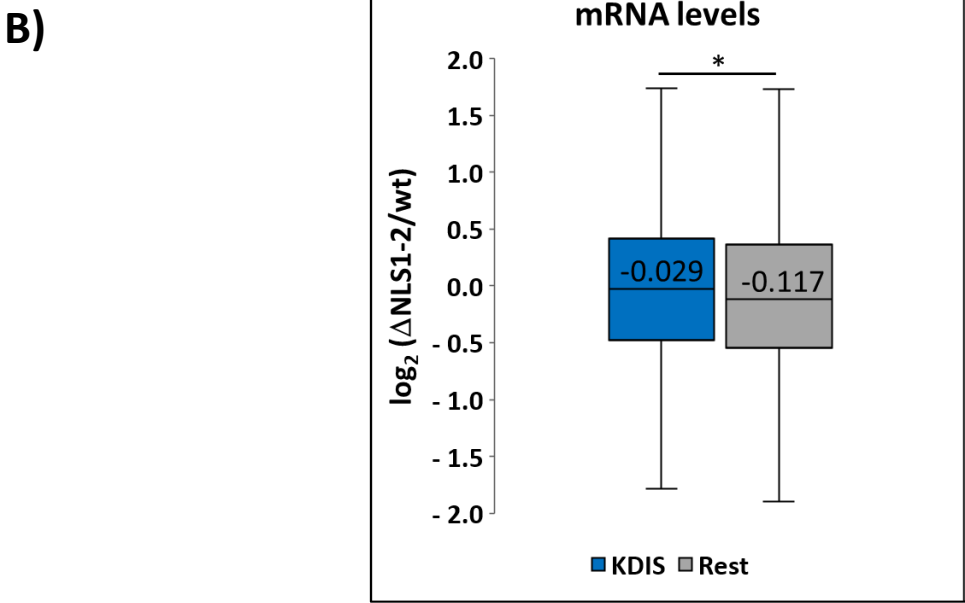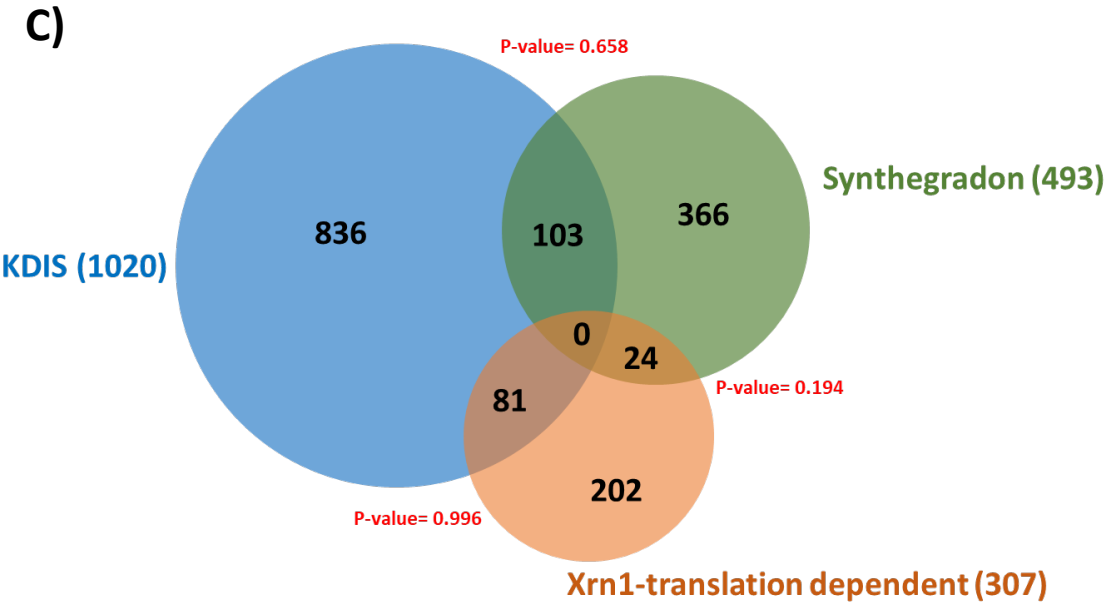

Supplementary Figure S2

Supplement: S2 Fig — A) Heat map displaying the measured poly(A) site reads for the wild-type (WT) and a xrn1Δ strain for KDIS and non KDIS genes. As expected, the bulk of the poly(A) site reads occur downstream of the stop codon, and a small fraction occurs prior to the start site (as expected from those originating from the upstream genes in tandem). KDIS genes do not display an increased number of poly(A) reads downstream of the start codon in the xrn1Δ strain. This suggests that XRN1 depletion does not lead to the accumulation of overlapping cryptic transcripts over the 5’region of KDIS. Plot generated from GSE40110 and GSE158548 using DeepTools [35]. Three individual replicates of WT and two of xrn1Δ strains are shown. B) Boxplots showing mRNA levels of KDIS vs. the rest of the genes (data from ref. [20]). The significance of the median comparisons was estimated using a Wilcoxon test: * = p< 0.05. C) Statistical study of KDIS genes. Venn diagrams show that KDIS genes do not significantly overlap those genes that depend more on Xrn1 for transcription activation and mRNA decay (synthegradon, Ref. [18]) and those genes whose mRNAs depend on Xrn1 for translation activation [19]. Statistical significance for every pair-wise comparison is indicated. (PDF) [file pone.0308195.s002.pdf]

Figure S3

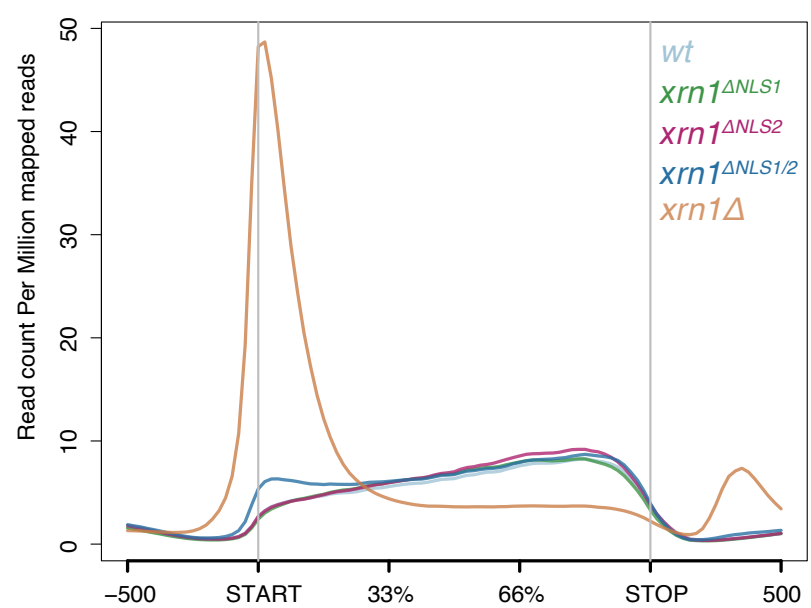

Supplement: S3 Fig — The same plot shown in Fig 1D showing the coverage in relation to the ORF start and stop codon for the wild-type (XRN1) with a version lacking NLS1 (xrn1ΔNLS1) or NLS2 (xrn1ΔNLS2), or both NLSs (xrn1ΔNLS1/2) is shown together an xrn1Δ sample from another study [21] normalized for the total reads added as a reference of a strain with no cytoplasmic 5’→3’ exoribonuclease activity. Note that the Y scales of this figure and Fig 1D are different. (PDF) [file pone.0308195.s003.pdf]

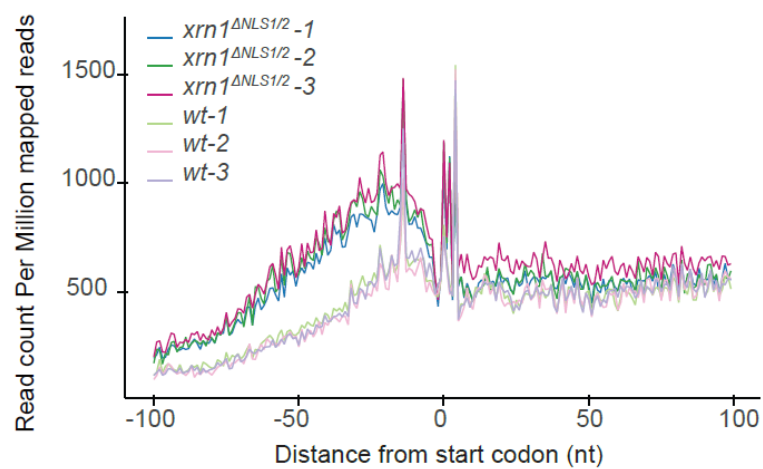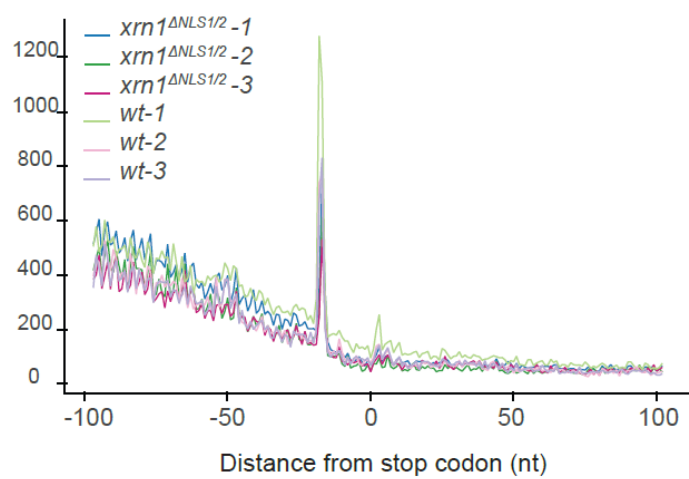

**Supplementary Figure S4**

Supplement: S4 Fig — High-resolution metagene analysis for the HT-5Pseq read coverage in individual three replicates of the wild-type (XRN1) or lacking both NLSs (xrn1ΔNLS1/2). The averaged plots are shown in Fig 1A–1C. Note the similarity of the replicates for each sample and the differences between wild type and xrn1ΔNLS1/2. (PDF) [file pone.0308195.s004.pdf]
